# Multi-Gene Phylogenetic Analyses Revealed Five New Species and Two New Records of *Distoseptisporales* from China

**DOI:** 10.3390/jof8111202

**Published:** 2022-11-14

**Authors:** Jian Ma, Jing-Yi Zhang, Xing-Juan Xiao, Yuan-Pin Xiao, Xia Tang, Saranyaphat Boonmee, Ji-Chuan Kang, Yong-Zhong Lu

**Affiliations:** 1 School of Food and Pharmaceutical Engineering, Guizhou Institute of Technology, Guiyang 550003, China; 2 Center of Excellence in Fungal Research, Mae Fah Luang University, Chiang Rai 57100, Thailand; 3 Guizhou Key Laboratory of Agricultural Biotechnology, Guizhou Academy of Agricultural Sciences, Guiyang 550006, China; 4 School of Science, Mae Fah Luang University, Chiang Rai 57100, Thailand; 5 Engineering and Research Center for Southwest Bio-Pharmaceutical Resources of National Education Ministry of China, Guizhou University, Guiyang 550025, China

**Keywords:** new taxa, *Aquapteridospora*, *Distoseptispora*, hyphomycete, submerged wood, taxonomy

## Abstract

Eight hyphomycetes were collected as part of an investigation into the diversity of hyphomycetous fungi in China. Based on morphology and multi-loci (LSU, ITS, *tef1α*, and *rpb2*) phylogenetic analyses, five new taxa, including a new *Aquapteridospora* species *A*. *hyalina* and four novel *Distoseptispora* species, *viz D*. *aquisubtropica*, *D*. *septata*, *D*. *tropica,* and *D*. *wuzhishanensis* were introduced in *Distoseptisporales* (*Sordariomycetes*). Two new habitat records, *viz Distoseptispora pachyconidia* and *D*. *xishuangbannaensis* were firstly reported. Also provided in this study are detailed descriptions of eight new collections and a revised phylogenetic tree for the *Distoseptisporales*.

## 1. Introduction

The two genera, *Distoseptispora* (*Distoseptisporaceae*) and *Aquapteridospora* (*Aquapteridosporaceae*) were recently introduced to *Distoseptisporales* (*Diaporthomycetidae*) [1,2,3]. Yang et al. [4] established *Aquapteridospora* (*Diaporthomycetidae*) as a monotypic genus, with *A. lignicola* as the type species. In later research, Hyde et al. [1] introduced *Aquapteridosporaceae* to accommodate *Aquapteridospora* and placed this family in the order *Distoseptisporales* based on divergence estimates, morphological characteristics, and phylogenetic analysis. This was followed in the “Outline of Fungi and fungus-like taxa” by Wijayawardene et al. [5]. Currently, *Aquapteridospora* consists of five species, most of which were collected from freshwater habitats. Only, *A. bambusinum* was discovered on dead bamboo culms [6]. The genus is distinguished by its macronematous, mononematous, solitary, unbranched, cylindrical conidiophores; its polyblastic, pale brown conidiogenous cells; and its fusiform, euseptate, guttulate conidia [1,4]. According to phylogenetic analyses, *Aquapteridospora* formed a distinct sister clade to all *Distoseptispora* species [4,7]. Morphologically, *Aquapteridospora* has macronematous, mononematous and unbranched conidiophores, monoblastic conidiogenous cells, and solitary conidia, similar to *Distoseptispora*. However, *Aquapteridospora* differs from *Distoseptispora* in its terminal conidiogenous cells, longer conidiophores, and fusiform euseptate conidia [2,3,4,8].

The type species of *Distoseptispora* was designated as *D*. *fluminicola* by Su et al. [9]. In recent years, a number of *Distoseptispora* (*Distoseptisporaceae*, *Distoseptisporales*) species have been accepted. Currently, 55 species including 41 freshwater species and 14 terrestrial species have been accepted in *Distoseptispora* [2,10,11,12,13,14,15,16]. Most *Distoseptispora* species are located in Asia, primarily in China and Thailand, with 26 species in the former and 27 in the latter [3,17,18,19,20,21], while two species *viz D*. *adscendens* and *D*. *leonensis* [22,23] were found in Hungary and Malaysia, respectively. Most *Distoseptispora* species are saprobic on palms, *Pandanus*, *Tectona*, bamboo, *Clematidis*, and *Carex* including unidentified submerged wood [9,24,25,26,27,28], and only *D. caricis* has been reported as an endophytic species [29]. In addition, *D. hyalina* is the only one species known to have a sexual morph [7].

During a survey of hyphomycetes in China, eight hyphomycetous taxa were collected from Hainan and Guizhou Provinces. Based on morphological evidence and phylogenetic analyses of sequence combinations of LSU, ITS, *tef1α*, and *rpb2*, five new species in genera *Aquapteridospora* and *Distoseptispora*, namely *A*. *hyalina, D*. *aquisubtropica*, *D*. *septata*, *D*. *tropica,* and *D*. *wuzhishanensis,* and two new habitat records of *D*. *pachyconidia* and *D*. *xishuangbannaensis*, were identified, and their full descriptions and illustrations are provided in the present report.

## 2. Materials and Methods

### 2.1. Sample Collection, Specimen Examination, and Isolation

**Figure 1:**
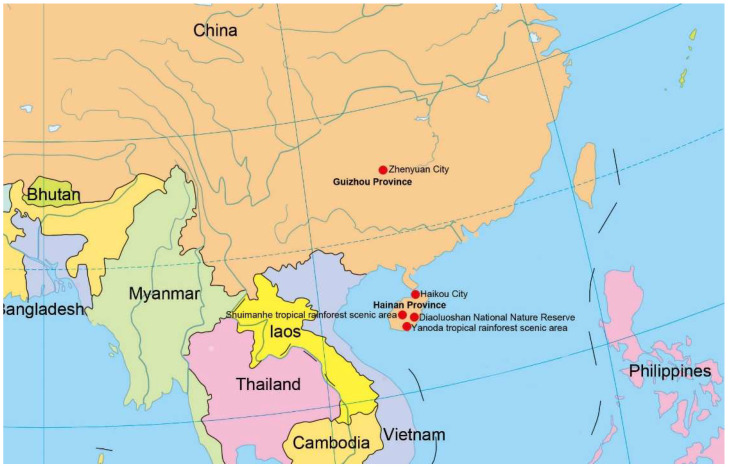
Collecting sites in this study (red dots).

Fresh specimens of decaying wood were randomly collected from freshwater and terrestrial habitats in Hainan and Guizhou Province, China (Figure 1). Samples were brought back to the laboratory in plastic bags with the collection details including localities and dates. Samples were incubated at room temperature in ziplock bags or sterile moist plastic boxes for about two weeks. Colonies on decaying wood surface were examined, observed, and photographed for their appearance with stereomicroscopes (SMZ 745 and SMZ 800N, Nikon, Tokyo, Japan) from low (0.75 times) to high (5 times) magnification. Fresh colonies were picked with sterile needles at a stereomicroscope magnification of 5 times and placed on a slide with a small amount of distilled water, and then placed under a Nikon EOS 90D digital camera attached to ECLIPSE Ni compound microscope (Nikon, Tokyo, Japan) for microscopic morphological characteristics. The dimensions of conidiophores, conidiogenous cells, and conidia were measured using Tarosoft (R) Image Frame Workprogram. In the species descriptions, arithmetic means as “$\bar{x}$”, and “n” stand for the number of measured elements. Photoplates were processed with Adobe PhotoShop CC 2019 (Adobe Systems, San Jose, CA, USA).

Single spore isolations were performed on water agar (WA) and germinated conidia were aseptically transferred to fresh potato dextrose agar (PDA) following the method of Senanayake et al. [30]. Cultures were grown on PDA and incubated in an incubator at 25 °C for 5 weeks and morphological characters, including color, shape, and size were recorded. The dried specimens were deposited at the Herbarium of Kunming Institute of Botany, Chinese Academy of Sciences (HKAS), Kunming, China, and the Herbarium of Guizhou Academy of Agriculture Sciences (GZAAS), Guiyang, China. Cultures were deposited in Guizhou Culture Collection, China (GZCC). Index Fungorum and Faces of Fungi numbers were acquired by the guideline in Jayasiri et al. [31] and Index Fungorum (2022) [32].

### 2.2. DNA Extraction, PCR Amplification, and Sequencing

Fresh fungal mycelia were scraped with sterilized toothpicks and transferred to 1.5 mL microcentrifuge tubes. Genomic DNA was extracted using the Biospin Fungus Genomic DNA Extraction Kit (BioFlux, Shanghai, China), following the manufacturer’s protocol. The primer pairs of LR0R/LR5, ITS5/ITS4, 983F/2218R, and frpb2-5f/frpb2-*7*cr were used to amplify the large subunit ribosomal DNA (LSU) [33], the internal transcribed spacer (ITS) [34], the translation elongation factor 1 alpha (*tef1α*) [35] and the RNA polymerase II second largest subunit (*rpb2*) gene regions [36], respectively. The amplification reactions were completed in a 50 μL reaction volume, including 2 μL DNA template, 2 μL of each forward and reverse primers, and 44 μL of 1.1 × T3 Supper PCR Mix (Qingke Biotech, Chongqing, China). Amplification reactions were carried out as follows (Table 1).

The quality of PCR amplification products was verified on 1% agarose electrophoresis gels stained with ethidium bromide. Purification and sequencing of PCR products were completed at Beijing Tsingke Biological Engineering Technology and Services Co., Ltd. (Beijing, China).

### 2.3. Phylogenetic Analyses

Original sequences were checked using BioEdit v 7.0.5.3 [37]. Forward and reverse sequences were assembled using SeqMan v. 7.0.0 (DNASTAR, Madison, WI, USA). The taxa used in this study were selected based on the closest matches from BLASTn search results (https://blast.ncbi.nlm.nih.gov/Blast.cgi, accessed on 15 October 2022), and from previous studies (Table 2) [7,12,28]. Sequence alignments for each locus were performed using the online multiple alignment program MAFFT version 7 (https://mafft.cbrc.jp/alignment/server/, accessed on 20 October 2022) [37,38], and auto-adjusted by trimAl tool [39]. A phylogenetic tree, which infers a phylogenetic relationship, was reconstructed based on a concatenated LSU, ITS, *tef1α*, and *rpb2* dataset using the online CIPRES Science Gateway (https://www.phylo.org/portal2/home.action, accessed on 29 October 2022) and analyzed using Maximum Likelihood (ML), Maximum Parsimony (MP) and Bayesian Inference (BI).

The “ALTER” (http://www.sing-group.org/ALTER/, accessed on 29 October 2022) website was used to convert the aligned fasta file to the phylip format for ML analyses [40]. ML analyses were performed through the CIPRES science Gateway V. 3.3 (https://www.phylo.org/portal2/home.action, accessed on 29 October 2022) [41]. The ML analysis was carried out using the RAxML-HPC v.8 on XSEDE (8.2.12) tool using a GTRGAMMA approximation with rapid bootstrap analysis followed by 1000 bootstrap replicates [42]. Maximum Parsimony (MP) analysis was carried out by using PAUP on XSEDE (4.a168) online website [43]. The 1000 random taxa were added for a heuristic search to infer MP trees. The value of MaxTrees, which collapsed branches of zero length and saved all multiple parsimonious trees, was set to 5000. Parsimony score values of tree length (TL), consistency index (CI), retention index (RI), and homoplasy index (HI) were calculated for trees generated under different optimum criteria. Clade stability was estimated using a bootstrap analysis with 1000 replicates, and the taxa was added for the random stepwise of each with 10 replicates [44].

The aligned fasta file was converted to the nexus format file for BI analysis by using AliView v. 1.27 [40]. Bayesian Inference (BI) analyses were performed by using MrBayes on XSEDE (3.2.7a) via CIPRES [42]. The best-fit evolutionary model for the individual and combined datasets was determined using MrModeltest v. 2.3. 10 [45]. GTR + G + I substitution model was selected for LSU, ITS, *tef1α*, and *rpb2*. The posterior probabilities (PP) were determined based on Bayesian Markov chain Monte Carlo (BMCMC) sampling [46]. Four simultaneous Markov chains were run for 10,000,000 generations, and trees were sampled every 1000th generation (resulting in 10,000 trees). The first 2500 trees, which represented the burn-in phase of the analysis, were discarded. The posterior probabilities (PP) in the majority rule consensus tree were calculated by the remaining 7500 trees.

Phylogenetic trees were visualized with FigTree v. 1.4.4. Adobe PhotoShop CC 2019 (Adobe Systems, San Jose, CA, USA) and Adobe Illustrator CC 2019v. 23.1.0 (Adobe Systems, San Jose, CA, USA) were used to edit trees and figures layout. Sequences generated in this study were deposited in GenBank (Table 2).

## 3. Phylogenetic Results

**Figure 2:**
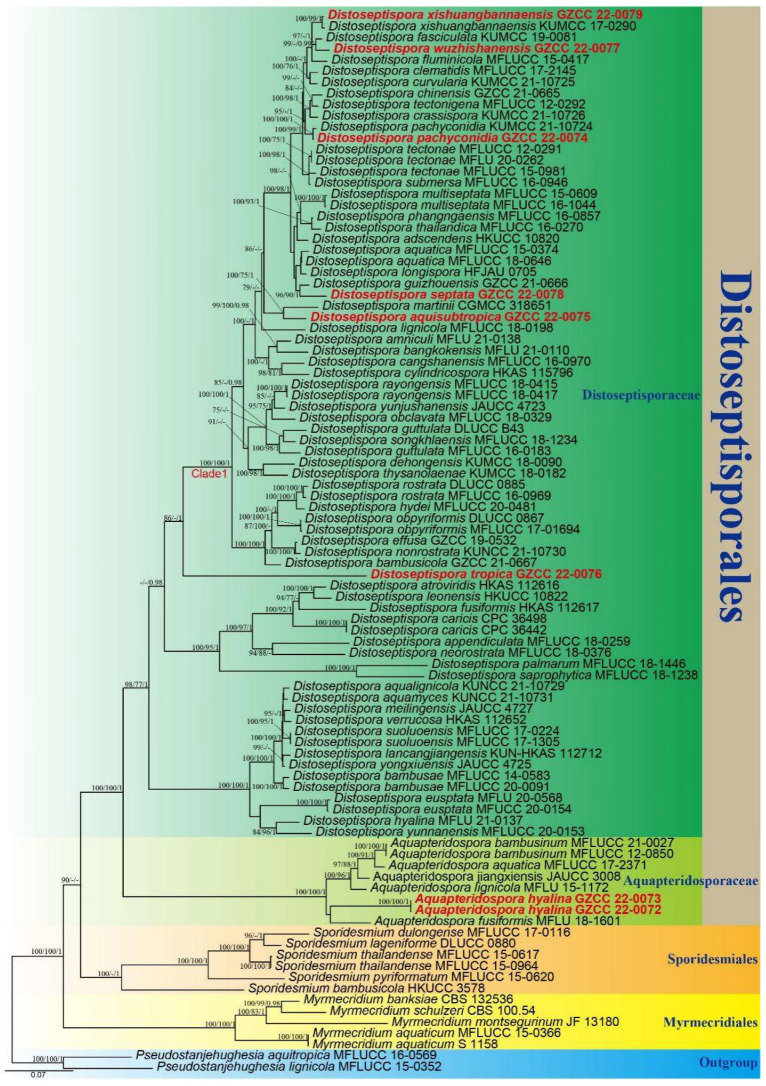
Phylogenetic tree generated from maximum likelihood (ML) analysis based on a combined of LSU, ITS, *tef1α*, and *rpb2* sequence data. Bootstrap support values of maximum likelihood (ML) and Maximum Parsimony (MP) equal to or greater than 75%, and Bayesian posterior probabilities (PP) equal to or greater than 0.95 are given near the nodes as ML/MP/PP. T *Pseudostanjehughesia aquitropica* (MFLUCC 16-0569) and *Ps*. *lignicola* (MFLUCC 15-0352) were used as outgroup taxa. The newly generated sequences are indicated in red bold.

Using partial nucleotide sequences from four genes, the phylogenetic placements of our new collections were determined. The concatenated sequence matrix comprised LSU (1–855 bp), ITS (856–1448 bp), *tef1α* (1449–2367 bp), and *rpb2* (2368–3420 bp) with a total of 3420 characters for 93 taxa and two outgroups (*Pseudostanjehughesia aquitropica* MFLUCC 16-0569 and *Ps*. *lignicola* MFLUCC 15-0352) [7,12,16,47,48,49,50]. Four gene analyses were conducted to compare the respective topologies and clade stabilities. There were 1733 distinct alignment patterns in the matrix, along with 31.09% undetermined characters or gaps. The ML, MP, and BI analyses of the concatenated LSU, ITS, *tef1α*, and *rpb2* dataset yielded similar tree topologies, and Figure 2 depicts the final likelihood value of the ML analysis.

The phylogenetic tree (Figure 2) demonstrates that our eight collections represent seven species within *Distoseptisporales*. Two isolates (GZCC 22-0072 and GZCC 22-0073) represent the new species *Aquapteridospora hyalina* which forms a distinct clade from other taxa of *Aquapteridospora* with strong support. *Distoseptispora aquisubtropica* and *D*. *martinii* are distinguished by their distinct conidial characteristics. *Distoseptispora septata* forms a sister lineage to *D*. *guizhouensis* with well support (96% ML/90% MP/1 PP). *Distoseptispora tropica* (GZCC 22-0076) is a significantly distinct lineage from other taxa (Figure 2). *Distoseptispora wuzhishanensis* forms a sister lineage to *D*. *fasciculata* with well support (97% ML/1 PP). Our isolate GZCC 22-0079 is recognized as *D*. *xishuangbannaensis* and GZCC 22-0074 as *D*. *pachyconidia*, and their molecular data are provided, respectively.

## 4. Taxonomy

**Figure 3:**
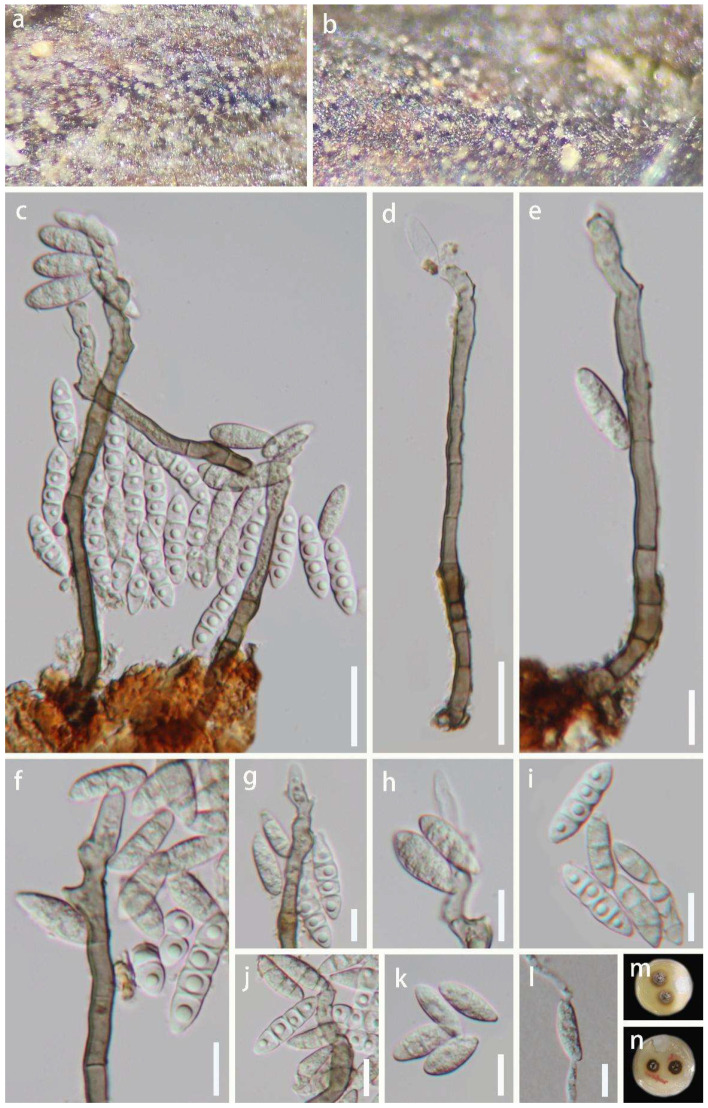
*Aquapteridospora hyalina* (HKAS 123764, holotype). (**a**,**b**) Colonies on the host surface. (**c**–**e**) Conidiophores, conidiogenous cells with attached conidia. (**f**–**h**) Conidiogenous cells bearing conidia. (**i**–**k**) Conidia. (**l**) Germinated conidium. (**m**,**n**) Colonies on PDA, m from above, n from below. Scale bars: (**e**–**l**) 20 μm, (**c**,**d**) 10 μm.

*Aquapteridospora hyalina* J. Ma and Y.Z. Lu., sp. nov., Figure 3.

Index Fungorum number: IF559932; Facesoffungi number: FoF11002.

Etymology: The epithet ‘*hyalina*’ referring to the colourless conidia.

Holotype: HKAS 123764.

*Saprobic* on decaying wood in a freshwater habitat. **Sexual morph:** Undetermined. **Asexual morph:** *Colonies* on the natural substrate, effuse, solitary, hyaline. *Mycelium* mostly superficial, consisting of branched, septate, smooth, pale brown to brown hyphae. *Conidiophores* 68–130 × 4.5–6.5 μm ($\bar{x}$ = 91 × 5.5 μm, n = 30), macronematous, mononematous, solitary, erect, simple, straight, or slightly flexuous, unbranched, smooth, cylindrical, 3–7-septate, thick-walled, smooth-walled, brown at the base, subhyaline to pale brown towards apex. *Conidiogenous cells* 25–62 × 4–6.5 μm ($\bar{x}$ = 44 × 5 μm, n = 30), polyblastic, monoblastic, smooth-walled, terminal, sub-cylindrical or gradually tapering towards tip, sub-hyaline to pale brown, forming conidia sympodially on conspicuous denticles, bearing tiny, protuberant, circular scars. *Conidia* 17–28 × 4–6 μm ($\bar{x}$ = 20 × 5.5 μm, n = 50) acropleurogenous, solitary, fusiform, 1–3-septate, truncate obtuse at septum, hyaline when young, sub-hyaline to pale brown when mature, tapering and pointed at both ends, smooth-walled, often with single guttulate in each cell when young.

Culture characteristics: Conidia were germinated on water agar and produced germ tubes within 10 h. Colonies grown on PDA, circular, flat, reaching 18 mm diam. after 25 days of incubation at 25 °C, lightly gray at the entire margin, brown in the center, reverse-side pale brown or brown at the entire margin, pale grey in the center.

Material examined: China, Hainan Province, Baoting County, Sandao Town, Yanoda Rainforest cultural tourism area, 18°46′ N, 109°65′ E, on rotting wood in a freshwater stream, 23 October 2021, Jian Ma, Y6 (HKAS 123764, holotype; GZAAS 22-0077, isotype), ex-type living culture GZCC 22-0072; idem, Y10-1 (HKAS 123763), living culture GZCC 22-0073.

Notes: The proposed new species *Aquapteridospora hyalina* shares similar characteristics with other species of the genus *Aquapteridospora* in having macronematous, mononematous, unbranched, polyblastic conidiophores, sympodial proliferations conidiogenous cells, and fusiform and acropleurogenous conidia [4]. However, *A*. *hyalina* differs from all species of *Aquapteridospora* in having hyaline to pale brown conidia with distinguished guttulae in each cell when young. The phylogenetic analyses confirmed that the two strains (GZCC 22-0072 and GZCC 22-0073) of *A*. *hyalina* form a distinct clade sister to *A*. *fusiformis* within *Aquapteridosporaceae* (Figure 2). Therefore, based on the morphological evidence with multi-gene phylogenetic results, a new species *A*. *hyalina* is introduced.

**Figure 4:**
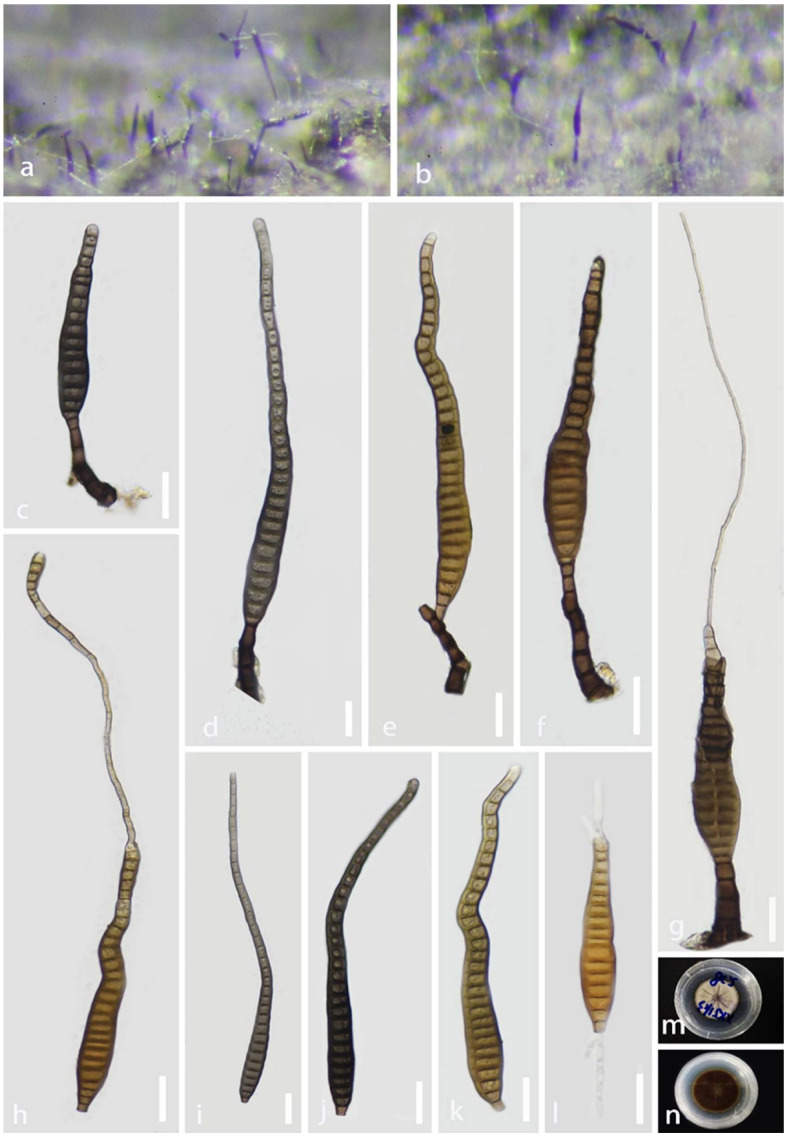
*Distoseptispora aquisubtropica* (GZAAS 22-0080, holotype). (**a**,**b**) Colonies on the host surface. (**c**–**g**) Conidiophores and conidiogenous cells bearing conidia. (**h**–**k**) Conidia. (**l**) Germinating conidium. (**m**,**n**) Colonies on PDA, m from above, n from below. Scale bars: (**c**–**l**) 20 μm.

*Distoseptispora aquisubtropica* J. Ma and Y.Z. Lu, sp. nov., Figure 4.

Index Fungorum number: IF559686; Facesoffungi number: FoF11334.

Etymology: ‘*aquisubtropica*’ is derived from subtropical, which means climate type and ‘aqui’ refers to its presence in aquatic habitat.

Holotype: HKAS 124023.

*Saprobic* on decaying wood submerged in a freshwater habitat. **Sexual morph:** Undetermined. **Asexual morph:** hyphomycetous. *Colonies* on natural substrate superficial, effuse, gregarious, smooth, septate, hairy, brown, or dark brown. *Mycelium* mostly immersed, composed of branched, septate, smooth, pale brown to brown hyphae. *Conidiophores* 16–83 × 5–11 μm ($\bar{x}\;$= 51 × 8 μm, n = 25), macronematous, mononematous, cylindrical, erect, simple, straight, or slightly flexuous, unbranched, smooth, thick-walled, solitary, brown at the base, pale brown or sub-hyaline towards the apex, 2–5-septate. *Conidiogenous cells* 3–11 × 3–7 μm ($\bar{x}$ = 6 × 5.5 μm, n = 25), holoblastic, monoblastic, terminal, integrated, cylindrical, pale brown or brown, smooth. *Conidia* 43–278 × 11–19 μm ($\bar{x}\;$= 141 × 14.5 μm, n = 38), acrogenous, solitary, multi-distoseptate, obclavate or lanceolate, rostrate, straight or slightly curved, verrucose, guttulate, thick-walled, smooth-walled, pale brown or dark brown, olivaceous, 16–31-distoseptate, usually paler towards apex, sometimes have conspicuous hyphae attached to the conidium, rounded at apex, with a truncate base.

Culture characteristics: Conidia were germinated on water agar and produced germ tubes within 10 h. Colonies grown on PDA, circular, flat, dense, dark gray, fluffy, reaching 34 mm diam. after 25 days of incubation at 25 °C, break in the center, from below olivaceous to brown at the center, pale yellow at the entire margin.

Material examined: China, Guizhou Province, Zhenyuan County, 27°05′ N, 108°41′ E, on decaying wood submerged from a freshwater stream, 1 May 2021, Jian Ma, XXJ11-3 (HKAS 124023, holotype; GZAAS 22-0080, isotype), ex-type living culture GZCC 22-0075.

Notes: According to a BLASTn search on NCBI GenBank, the ITS and LSU sequences of the new isolate (*Distoseptispora aquisubtropica*) share 92.95% similarity across 85% of the query sequence coverage and 99.6% similarity across 86% of the query sequence coverage with *D*. *martini*, respectively. The phylogenetic results indicate that *D*. *aquisubtropica* forms a sister clade to *D*. *martinii* with well-supported values (100% ML/75% MP/1 PP). The morphological features of *D*. *aquisubtropica* are compatible with the *Distoseptispora* generic concept, while *D*. *martini* resembles *Acrodictys* rather than *Distoseptospora* [13]. *Distoseptospora aquisubtropica* can be distinguished from *D*. *martini* by its obclavate or lanceolate conidia, while the latter is oblate or subglobose [13]. Based on a pairwise comparison of ITS and LSU nucleotides, *D*. *aquisubtropica* differs from *D*. *martinii* in 24/453 bp (5.3%) for ITS and 5/515 bp (0.97%) for LSU, respectively. Therefore, we identified *D*. *aquisubtropica* as a novel species, according to the species delimitation guidelines proposed by Chethana et al. and Maharachchikumbura et al. [51,52].

**Figure 5:**
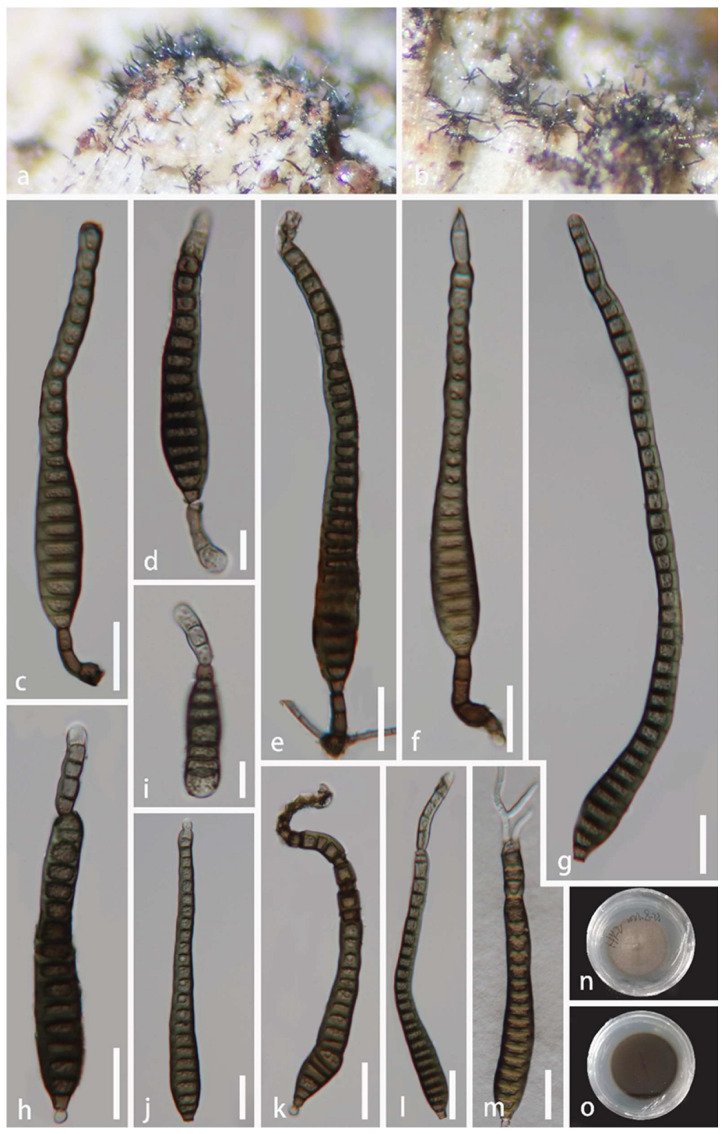
*Distoseptispora pachyconidia* (HKAS 123754). (**a**,**b**) Colonies on the host surface. (**c**–**f**) Conidiophores and conidiogenous cells bearing conidia. (**g**–**l**) Conidia. (**m**) Germinating conidium. (**n**,**o**) Colonies on PDA, n from above, o from reverse. Scale bars: (**c,e**–**h**,**j**–**m**) 20 μm, (**d**,**i**) 10 μm.

*Distoseptispora pachyconidia* R. Zhu and H. Zhang. Journal of Fungi 30: 22 (2022). Figure 5.

Index Fungorum Number: IF559924; Facesoffungi number: FoF12581.

*Saprobic* on decaying wood in terrestrial habitats. **Sexual morph:** Undetermined. **Asexual morph:** *Colonies* effuse, gregarious, brown, or dark brown, hairy. *Mycelium* immersed and partly superficial, consisting of branched, septate, smooth, pale brown to brown hyphae. *Conidiophores* 14–44 × 4–7 μm ($\bar{x}$ = 26 × 5 μm, n = 20), macronematous, mononematous, brown to dark brown, solitary, 2–4-septate, erect, straight, or flexuous, unbranched, smooth, cylindrical, singly or in groups, truncate at the apex, slightly constricted at septa. *Conidiogenous cells* 4–9 × 4–5.5 μm ($\bar{x}$ = 6 × 4.5 μm, n = 20), holoblastic, monoblastic, integrated, terminal, determinate, pale brown to brown, smooth, cylindrical. *Conidia* 50–242 × 11–20 μm ($\bar{x}$ = 111 × 13 μm, n = 30) acrogenous, solitary, obclavate, rostrate, smooth-walled, straight or slightly curved, up to 38-distoseptate, guttulate, olivaceous to dark brown, mostly slightly constricted at septa, tapering towards the rounded apex, truncate at the base.

Culture characteristics: Conidia were germinated on water agar and produced germ tubes within 10 h. Colonies grown on PDA, circular, mycelium flat, dense, reaching 40 mm diam. after 30 days of incubation at 25 °C, gray or brown, reverse-side dark brown.

Material examined: China, Hainan Province, Haikou City, Xiuying District, Ecological leisure trail, on decaying wood on the ground, 20°01′ N, 110°25′ E, 10 August 2021, Jian Ma, HK2 (HKAS 123754), living culture GZCC 22-0074.

Notes: Zhang et al. [16] introduced *Distoseptispora pachyconidia* from decaying wood in Yunnan Province, China. Analyses of multi-gene revealed that the new isolate GZCC 22-0074 clustered with *D*. *pachyconidia*. The morphological characteristics of this isolate are similar to the protologue of *D*. *pachyconidia*. However, GZCC 22-0074 differs from *D*. *pachyconidia* in having different colors of conidia (olivaceous to dark brown vs. pale-brown with a green tinge) and the number of conidial septa (up to 38 vs. 8–21-distoseptate) [16]. According to a pairwise nucleotide comparison of ITS, LSU, *tef1α* and *rpb2*, our isolate differs from the type strain of *D*. *pachyconidia* (HKAS 122179) in 1/520 bp (0.2%) for ITS, 1/852 bp (0.1%) for LSU, 1/913 bp (0.1%) for *tef1α* and 0/1052 bp (0%) for *rpb2*, respectively. Thus, the phylogenetic evidence did not show significant differences between them (Figure 2). We therefore identified the new isolate as *D*. *pachyconidia*.

**Figure 6:**
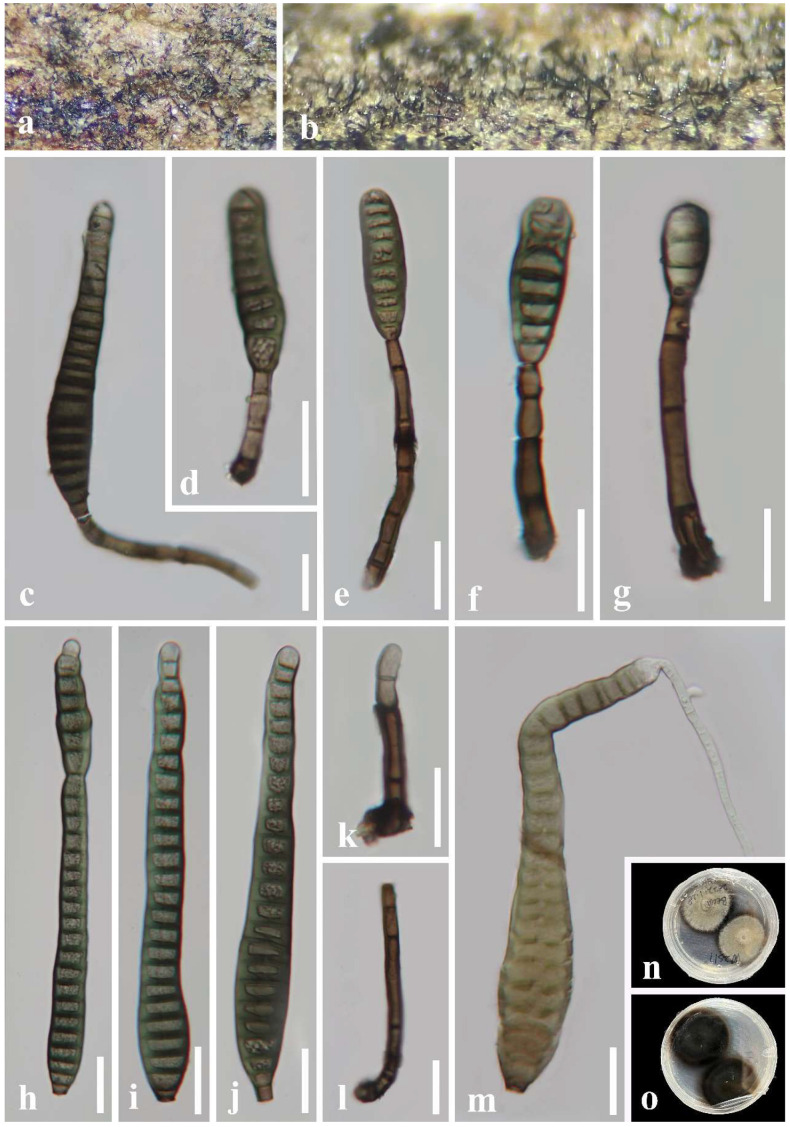
*Distoseptispora septata* (HKAS 123759, holotype). (**a**,**b**) Colonies on the host surface. (**c**–**g**) Conidiophores and conidiogenous cells bearing conidia. (**h**–**j**) Conidia. (**k**,**l**) Conidiophores and conidiogenous cells. (**m**) Germinating conidium. (**n**,**o**) Colonies on PDA, n from above, o from reverse. Scale bars: (**c**–**m**) 20 μm.

*Distoseptispora septata* J. Ma and Y.Z. Lu, sp. nov., Figure 6.

Index Fungorum number: IF559688; Facesoffungi number: FoF11337.

Etymology: ‘*septata*’ referring to ‘septate’ conidia.

Holotype: HKAS 123759.

*Saprobic* on decaying wood submerged in a freshwater habitat. **Sexual morph:** Undetermined. **Asexual morph:** hyphomycetous. *Colonies* on natural substrate superficial, effuse, gregarious, smooth, septate, hairy, brown, or dark brown. *Mycelium* mostly immersed, composed of branched, septate, smooth, pale brown to brown hyphae. *Conidiophores* 23–86 × 3–7 μm ($\bar{x}$ = 44 × 5.5 μm, n = 30), macronematous, mononematous, cylindrical, erect, simple, mostly flexuous, unbranched, smooth, thick-walled, solitary, brown, 1–6-septate. *Conidiogenous cells* 5–18 × 4–6 μm ($\bar{x}$ = 9 × 5 μm, n = 30), holoblastic, monoblastic, terminal, integrated, cylindrical, pale brown or brown, smooth. *Conidia* 22–179 × 10–16 μm ($\bar{x}$ = 89 × 13 μm, n = 35), acrogenous, solitary, multi-distoseptate and up to 25-septate, obclavate, rostrate, straight or slightly curved, verrucose, guttulate, thick-walled, smooth-walled, pale brown or dark brown, olivaceous-green, usually paler towards apex, rounded at apex, with a truncate base.

Culture characteristics: Conidia were germinated on water agar and produced germ tubes within 10 h. Colonies grown on PDA, circular, flat, dense, fluffy, reaching 35 mm diam. after 25 days of incubation at 25 °C, olivaceous-green, paler brown and dark brown, reverse-side dark brown.

Material examined: China, Hainan Province, Wuzhishan City, Shui Man Town, Wuzhishan National Nature Reserve, 18°92′ N, 109°63′ E, on dead wood in a freshwater stream, 26 December 2021, Xia Tang, W17 (HKAS 123759, holotype; GZAAS 22-0083, isotype), ex-type living culture GZCC 22-0078.

Notes: *Distoseptispora septata* shares a sister relationship to *D*. *guizhouensis* with strong support (96% ML/90% MP/1 PP). However, *D*. *septata* differs from *D*. *guizhouensis* in having smaller conidia (22–179 × 10–16 μm vs. 90–273 × 15–21 μm). Particularly, *D*. *guizhouensis* is distinguished from *D*. *septata* by distinctly flexuous conidiophores. Based on a pairwise comparison of ITS nucleotides, *D*. *septata* differs from *D*. *guizhouensis* by 13/535 bp (2.4%). Following the guidelines for defining species boundaries of Chethana et al. and Pem et al. [51,53], we therefore introduce GZCC 22-0078 as a new species.

**Figure 7:**
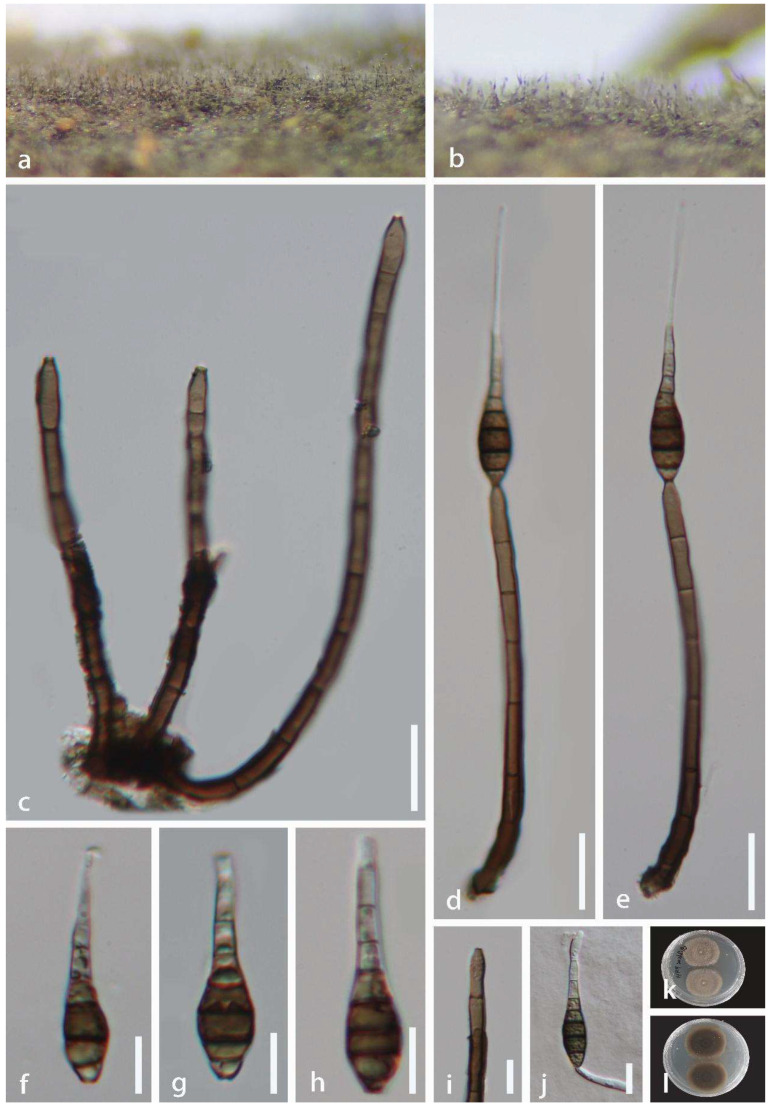
*Distoseptispora tropica* (HKAS 123761, holotype). (**a**,**b**) Colonies on the host surface. (**c**) Conidiophores and conidiogenous cells. (**d**,**e**) Conidiophores with attached conidia. (**f**–**h**) Conidia. (**i**) Conidiogenous cell. (**j**) Germinating conidium. (**k**,**l**) Colonies on PDA, k from above, l from reverse. Scale bars: (**c**–**e**) 20 μm, (**f**–**j**) 10 μm.

*Distoseptispora tropica* J. Ma and Y.Z. Lu, sp. nov., Figure 7.

Index Fungorum number: IF559689; Facesoffungi number: FoF11335.

Etymology: ‘*tropica*’ derived from the climate in which the species was discovered.

Holotype: HKAS 123761.

*Saprobic* on dead wood, in terrestrial habitat. **Sexual morph:** Undetermined. **Asexual morph:** hyphomycetous. *Colonies* on dead wood, effuse, scattered or in small groups, gregarious, hairy, smooth, brown, or dark brown. *Mycelium* mostly immersed, composed of branched, septate, smooth, brown to dark brown. *Conidiophores* 60–151 × 3.5–7 μm ($\bar{x}$ = 94 × 5 μm, n = 20), macronematous, mononematous, cylindrical, erect, straight, or slightly flexuous, unbranched, smooth, thick-walled, dark brown, solitary or caespitose, dark brown and rounded at the apex, paler brown at the upper part, 5–7 septate. *Conidiogenous cells* 10–21 × 3.5–6 μm ($\bar{x}$ = 159 × 5 μm, n = 20), holoblastic, monoblastic, terminal, integrated, cylindrical, pale brown or brown, smooth. *Conidia* 39–75 × 7.5–10.5 μm ($\bar{x}$ = 47 × 8 μm, n = 30), acrogenous, verrucose, solitary, multi-distoseptate, obclavate, rostrate, upper part tapering towards the apex, guttulate, thick-walled, smooth, olivaceous brown or dark brown, 5–7 distoseptate, with conspicuous hyphae attachment conidium, with a truncate base.

Culture characteristics: Conidia were germinated on water agar and produced germ tubes within 10 h. Colonies grown on PDA, circular, flat, dense, fluffy, reaching 40 mm diam. after 40 days of incubation at 25 °C, grayish brown mycelium on the surface, reverse-side dark brown.

Material examined: China, Hainan Province, Haikou City, Xiuying District, Ecological leisure trail, 20°01′ N, 110°25′ E, on decaying wood in terrestrial habitat, 10 August 2021, Jian Ma, HK9 (HKAS 123761, holotype; GZAAS 22-0081, isotype), ex-type living culture GZCC 22-0076. 

Notes: *Distoseptispora tropica* shares an extremely similar morphology with the species of *Distoseptispora*, *Ellisembia*, and *Sporidesmium* [7,9,54]. However, phylogenetic analysis (Figure 2) indicates that *D*. *tropica* belongs to *Distoseptispora*. *Distoseptispora tropica* forms a distinct lineage basal to Clade 1 with strong support (100% ML/100% MP/1 PP). However, *D*. *tropica* differs from other taxa in Clade 1 in having longer conidiophores and smaller conidia.

**Figure 8:**
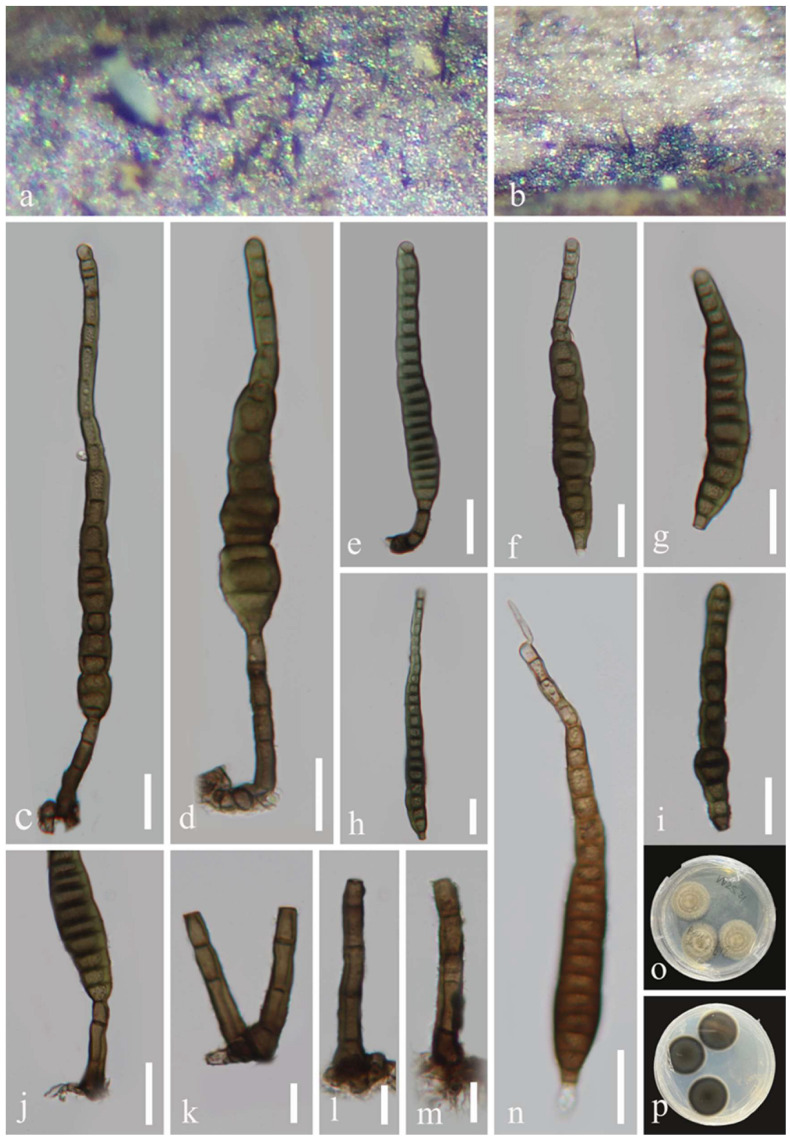
*Distoseptispora wuzhishanensis* (HKAS 123762, holotype). (**a**,**b**) Colonies on the host surface. (**c**–**e**) Conidiophores and conidia. (**f**–**i**) Conidia. (**j**–**m**) Conidiophores and conidiogenous cells. (**n**) Germinating conidium. (**o,p**) Colonies on PDA, o from above, p from reverse. Scale bars: (**c**–**j**,**n**) 20 μm, (**k**–**m**) 10 μm.

*Distoseptispora wuzhishanensis* J. Ma and Y.Z. Lu, sp. nov., Figure 8.

Index Fungorum number: IF559706; Facesoffungi number: FoF11336.

Etymology: ‘*wuzhishanensis*’ derived from the city where the species was discovered.

Holotype: HKAS 123762.

*Saprobic* on decaying wood in a freshwater habitat. **Sexual morph:** Undetermined. **Asexual morph:** hyphomycetous. *Colonies* on substrate superficial, effuse, solitary, smooth, septate, hairy, brown, or dark brown. *Mycelium* mostly immersed, composed of branched, septate, smooth, pale brown to brown hyphae. *Conidiophores* 16–56 × 5–7 μm ($\bar{x}$ = 35 × 6 μm, n = 25), macronematous, mononematous, cylindrical, erect, scattered or in small groups, straight or slightly flexuous, unbranched, smooth, thick-walled, brown at the base, pale brown towards the apex, 1–4 septate. *Conidiogenous cells* 6.5–10.5 × 4.5–6 μm ($\bar{x}$ = 8 × 5 μm, n = 25), holoblastic, monoblastic, terminal, integrated, cylindrical, pale brown or brown, smooth. *Conidia* 76–143 × 11–17 μm ($\bar{x}$ = 108 × 13.5 μm, n = 40), acrogenous, solitary, multi-distoseptate, obclavate, rostrate, straight, or slightly curved, verrucose, guttulate, thick-walled, smooth-walled, pale brown or dark brown, olivaceous-green and yellow, usually up to 22-distoseptate, strongly constricted at septa, usually paler towards apex, rounded at apex, with a truncate base.

Culture characteristics: Conidia were germinated on water agar and produced germ tubes within 10 h. Colonies grown on PDA, circular, flat, dense, fluffy, reaching 24 mm diam. after 25 days of incubation at 25 °C, olivaceous-green or brown mycelium on the surface, reverse side partly paler brown to dark brown.

Material examined: China, Hainan Province, Wuzhishan City, Shui Man Town, Wuzhishan National Nature Reserve, 18°92′ N, 109°63′ E, on dead wood in a freshwater stream, 26 December 2021, Xia Tang, W21 (HKAS 123762, holotype; GZAAS 22-0082, isotype), ex-type living culture GZCC 22-0077.

Notes: According to phylogenetic tree (Figure 2), *Distoseptispora wuzhishanensis* forms a close lineage to *D*. *fasciculata* with high bootstrap support (99% ML/0.99 PP). The morphological characteristics of the new taxon match well with the species concept of *Distoseptispora* in conidiophores and conidia. However, *D*. *wuzhishanensis* differs from *D*. *fasciculata* in having longer conidiophores (16–56 × 5–7 μm vs. 12–16 × 5–6 μm) [3]. 

**Figure 9:**
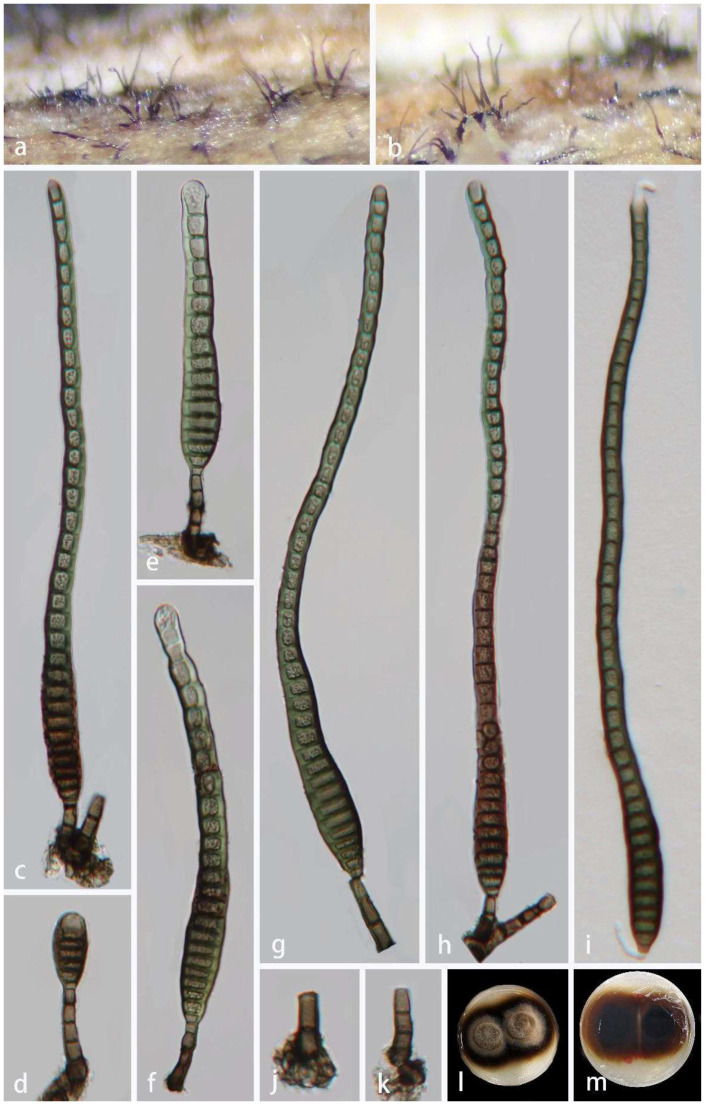
*Distoseptispora xishuangbannaensis* (HKAS 123760). (**a**,**b**) Colonies on the host surface. (**c**–**h**) Conidiophores, conidiogenous cells with attached conidia. (i) Germinating conidium. (**j**,**k**) Conidiophores and conidiogenous cells. (**l**,**m**) Colonies on PDA, l from above, m from below. Scale bars: (**c**,**k**,**e**–**i**) 20 μm, (**d**,**j**) 10 μm.

*Distoseptispora xishuangbannaensis* Tibpromma and K.D. Hyde. Fungal Diversity 93: 82 (2018). Figure 9.

Index Fungorum Number: IF554554; Facesoffungi number: FoF04563.

*Saprobic* on decaying wood, submerged in freshwater habitats. **Sexual morph:** Undetermined. **Asexual morph:** hyphomycetous. *Colonies* effuse, hairy, gregarious, olivaceous, or brown. *Mycelium* mostly immersed, composed of branched, smooth, septate, pale brown to brown hyphae. *Conidiophores* 19–52 × 5–7 μm ($\bar{x}$ = 29 × 6 μm, n = 20), macronematous, mononematous, unbranched, solitary, straight or slightly flexuous, brown at the base, pale brown towards the apex, slightly tapering distally, truncate at the apex, 1–4 septate. *Conidiogenous cells* 2–8.5 × 4.0–7 μm ($\bar{x}\;$= 6 × 5 μm, n = 20), blastic, holoblastic, monoblastic, terminal, integrated, cylindrical, pale brown. *Conidia* 88–269 × 11–15 μm ($\bar{x}$ = 165 × 13 μm, n = 30), solitary, acrogenous, cylindric-obclavate, rostrate, straight, or slightly curved, guttulate, thick-walled, smooth-walled, green-brown or brown, up to 42-distoseptate, usually paler olivaceous towards apex, rounded at apex, with a truncate base.

Culture characteristics: Conidia were germinated on water agar and produced germ tubes within 10 h. Colonies grown on PDA, circular, fluffy, reaching 36 mm diam. after 28 days of incubation at 25 °C, lightly grey in the center, olivaceous at the entire margin, reverse side pale brown and brown mycelium.

Material examined: China, Hainan Province, Lingshui Lizu Autonomous County, Diaoluoshan National Nature Reserve, 18°43′ N, 109°43′ E, on rotting wood in a freshwater habitat, 24 August 2021, Jian Ma, DL40 (HKAS 123760), living culture GZCC 22-0079.

Notes: Tibpromma et al. [20] introduced *Distoseptispora xishuangbannaensis* from dead leaf sheaths of *Pandanus utilis* in China. Analyses of multiple genes revealed that our new isolate (GZCC 22-0079) is closely related to *D*. *xishuangbannaensis. Distoseptispora xishuangbannaensis* possesses macronematous, straight or slightly flexuous conidiophores, holoblastic, monoblastic, brown conidiogenous cells, and acrogenous, cylindrical-obclavate conidia. The morphological characteristics of our isolate share similar characters with the holotype of *D*. *xishuangbannaensis* (HKAS 101809), but our isolate has longer and wider conidiophores (19–52 × 5–7 μm vs. 12–17 × 2–5 μm) and smaller conidia (88–269 × 11–15 μm vs. 160–305 × 8–15 μm) [20]. According to a pairwise nucleotide comparison of ITS, *tef1α* and *rpb2*, our new isolate differs from *D*. *xishuangbannaensis* in 5/498 bp (1%) for ITS, 3/898 bp (0.3%) for *tef1α* and 2/1052 bp (0.2%) for *rpb2*, respectively, and the phylogenetic result did not show significant differences between the new isolate and *D*. *xishuangbannaensis* (Figure 2). We therefore identified the new isolate as *D*. *xishuangbannaensis*.

## 5. Discussion

In recent years, studies on the number of saprotrophic fungi have received extensive attention [55,56]. For example, freshwater fungi occur in streams and other aquatic bodies [2,57], Calabon et al. [51] listed 3870 species, and numerous taxa are still being discovered [1]. Calabon et al. [51] listed 22 *Distoseptispora* species from freshwater and this study brings the total to 40 species. Analyses of morphological characteristics and molecular data indicate that eight collections, including five new species, namely *A*. *hyalina*, *D*. *aquisubtropica*, *D*. *septata*, *D*. *tropica,* and *D*. *wuzhishanensis* are introduced. Five *Aquapteridospora* epithets (*A*. *lignicola*, *A*. *fusiformis*, *A*. *bambusinum*, *A*. *jiangxiensis*, and *A*. *aquatica*) are listed in Index Fungorum [32,58]. Morphologically, *A*. *hyalina* corresponds well with the generic concept of *Aquapteridospora* [4,6,58]. However, the new isolate differs from other new species of genus *Aquapteridospora* by having conidia that have a truncate obtuse at their septum. Multi-gene analyses indicated that *A*. *hyalina*, a species phylogenetically distinct in the genus *Aquapteridospora*, was most closely related to *A*. *fusiformis* with weak support.

Morphologically, the asexual morph of *Distoseptispora* resembles *Sporidesmium* taxa [9]. Yang et al. [7] reported the existence of the sexual morph of *Distoseptispora*. Combining the morphological characteristics and the phylogenetic evidence of all species in genus *Distoseptispora*, we found that some *Distoseptispora* species form sister clades in phylogeny but have different morphologies. For example, *D. martini* and *D*. *aquisubtropica* have a close phylogenetic relationship. However, *D*. *martini* has distinctive oblate or subglobose conidia, while *D*. *aquisubtropica* has obclavate or lanceolate conidia. In addition, there are some species with similar morphologies but are genetically unrelated. For example, *D*. *tropica* and *D*. *verrocosa* have an identical morphology of conidiophores and conidia, but the two species have a distant phylogenetic relationship. Considering this phenomenon, additional molecular data and morphological characteristics are required for verification and expansion.

Previous studies of *Distoseptispora* have primarily been conducted in China (Guizhou, Yunnan, Sichuan, and Jiangxi Provinces) and in Thailand, which are subtropical and tropical regions. In this paper, eight collections were discovered in freshwater and terrestrial habitats in the Provinces of Hainan and Guizhou, China. Thus, it is unclear whether it has a close relationship with the climate. It may be a result of the limited geographical regions sampled.

## Figures and Tables

**Table 1:** PCR protocols.

Locus	Primer	Initial Denaturation	Denaturation	Annealing	Elongation	Final Extension	Hold
LSU	ITS5/ITS4	94 °C/3 min	94 °C/45 s	56 °C/50 s	72 °C/1 min	72 °C/10 min	4 °C
			40 cycles				
ITS	LR0R/LR5	94 °C/3 min	94 °C/45 s	56 °C/50 s	72 °C/1 min	72 °C/10 min	
			40 cycles				
*tef1α*	983F/2218R	94 °C/3 min	94 °C/30 s	56 °C/50 s	72 °C/1 min	72 °C/10 min	
			30 cycles				
*rpb2*	frpb2-5f/frpb2-*7*cr	95 °C/5 min	95 °C/15 s	56 °C/50 s	72 °C/1 min	72 °C/10 min	
			35 cycles				

**Table 2:** Taxa used in this study and the GenBank accession numbers of DNA sequences.

Taxon	Strain	GenBank Accessions				Reference
		LSU	ITS	*tef1α*	*rpb2*	
*Aquapteridospora* *fusiformis*	MFLU 18-1601^T^	MK849798	MK828652	MN194056	–	Luo et al. (2019)
*A*. *aquatica*	MFLUCC 17-2371^T^	MW287767	MW286493	–	–	Dong et al. (2021)
*A*. *bambusinum*	MFLUCC 12-0850^T^	KU863149	KU940161	KU940213		Dai et al. (2016)
*A*. *bambusinum*	MFLUCC 21-0027	MZ412526	MZ412514	MZ442688	–	Bao et al. (2021)
***A*. *hyalina***	**GZCC 22-0072^T^**	**ON527945**	**ON527937**	**ON533681**	**ON533689**	**This study**
***A*. *hyalina***	**GZCC 22-0073**	**ON527948**	**ON527940**	**ON533684**	**ON533691**	**This study**
*A*. *jiangxiensis*	JAUCC 3008^T^	MZ871501	MZ871502	MZ855767	MZ855768	Peng et al. (2022)
*A*. *lignicola*	MFLU 15-1172^T^	KU221018	MZ868774	MZ892980	MZ892986	Yang et al. (2015)
*Distoseptispora* *adscendens*	HKUCC 10820	DQ408561	–	–	DQ435092	Shenoy et al. (2006)
*D*. *amniculi*	MFLU 21-0138^T^	MZ868761	MZ868770	–	MZ892982	Yang et al. (2021)
*D*. *appendiculata*	MFLUCC 18-0259^T^	MN163023	MN163009	MN174866	–	Luo et al. (2019)
*D*. *aqualignicola*	KUNCC 21-10729^T^	ON400845	OK341186	OP413480	OP413474	Zhang et al. (2022)
*D*. *aquamyces*	KUNCC 21-10731^T^	OK341199	OK341187	OP413482	OP413476	Zhang et al. (2022)
*D*. *aquatica*	MFLUCC 15-0374^T^	KU376268	MF077552	–	–	Su et al. (2016)
*D*. *aquatica*	MFLUCC 18-0646	MK849793	MK828648	–	–	Luo et al. (2019)
***D*. *aquisubtropica***	**GZCC 22-0075^T^**	**ON527941**	**ON527933**	**ON533677**	**ON533685**	**This study**
*D*. *atroviridis*	HKAS 112616^T^	MZ868763	MZ868772	MZ892978	MZ892984	Yang et al. (2021)
*D*. *bambusae*	MFLUCC 20-0091^T^	MT232718	MT232713	MT232880	MT232881	Sun et al. (2020)
*D*. *bambusae*	MFLUCC 14-0583	MT232717	MT232712	–	MT232882	Sun et al. (2020)
*D*. *bambusicola*	GZCC 21-0667^T^	MZ474872	MZ474873	–	–	
*D*. *bangkokensis*	MFLU 21-0110^T^	MZ518206	MZ518205	–	–	Shen et al. (2021)
*D*. *cangshanensis*	MFLUCC 16-0970^T^	MG979761	MG979754	MG988419	–	Luo et al. (2018)
*D*. *caricis*	CPC 36498^T^	MN567632	MN562124	–	MN556805	Crous et al. (2019)
*D*. *caricis*	CPC 36442	–	MN562125	–	MN556806	Crous et al. (2019)
*D*. *chinensis*	GZCC 21-0665^T^	MZ474867	MZ474871	MZ501609	–	Hyde et al. (2021)
*D*. *clematidis*	MFLUCC 17-2145^T^	MT214617	MT310661	–	MT394721	Phukhamsakda et al. (2020)
*D*. *crassispora*	KUMCC 21-10726^T^	OK341196	OK310698	OP413479	OP413473	Zhang et al. (2022)
*D*. *curvularia*	KUMCC 21-10725^T^	OK341195	OK310697	OP413478	OP413472	Zhang et al. (2022)
*D*. *cylindricospora*	HKAS 115796^T^	OK513523	OK491122	OK524220	–	Phukhamsakda et al. (2022)
*D*. *dehongensis*	KUMCC 18-0090^T^	MK079662	MK085061	MK087659	–	Hyde et al. (2019)
*D*. *effusa*	GZCC 19-0532^T^	MZ227224	MW133916	–	–	Yang et al. (2021)
*D*. *eusptata*	MFUCC 20-0154^T^	MW081544	MW081539	–	MW151860	Li et al. (2021)
*D*. *eusptata*	MFLU 20-0568	MW081545	MW081540	MW084994	MW084996	Li et al. (2021)
*D*. *fasciculata*	KUMCC 19-0081^T^	MW287775	MW286501	MW396656	–	Dong et al. (2021)
*D*. *fluminicola*	MFLUCC 15-0417^T^	KU376270	MF077553	–	–	Su et al. (2016)
*D*. *fusiformis*	HKAS 112617^T^	MZ868764	MZ868773	MZ892979	MZ892985	Yang et al. (2021)
*D*. *guizhouensis*	GZCC 21-0666^T^	MZ474869	MZ474868	MZ501610	MZ501611	Hyde et al. (2021)
*D*. *guttulata*	MFLUCC 16-0183^T^	MF077554	MF077543	MF135651	–	Yang et al. (2018)
*D*. *guttulata*	DLUCC B43	MN163016	MN163011	–	–	Luo et al. (2019)
*D. hyalina*	MFLU 21-0137^T^	MZ868760	MZ868769	MZ892976	MZ892981	Yang et al. (2021)
*D*. *hydei*	MFLUCC 20-0481^T^	MT742830	MT734661	–	MT767128	Monkai et al. (2020)
*D*. *lancangjiangensis*	KUN-HKAS 112712^T^	MW879522	MW723055	–	MW882260	Shen et al. (2021)
*D*. *leonensis*	HKUCC 10822	DQ408566	–	–	DQ435089	Shenoy et al. (2006)
*D*. *lignicola*	MFLUCC 18-0198^T^	MK849797	MK828651	–	–	Luo et al. (2019)
*D*. *longispora*	HFJAU 0705^T^	MH555357	MH555359	–	–	Yang et al. (2021)
*D*. *martinii*	CGMCC 318651^T^	KX033566	KU999975	–	–	Xia et al. (2017)
*D*. *meilingensis*	JAUCC 4727^T^	OK562396	OK562390	OK562408	–	Zhai et al. (2022)
*D*. *multiseptata*	MFLUCC 16-1044	MF077555	MF077544	MF135652	MF135644	Yang et al. (2018)
*D*. *multiseptata*	MFLUCC 15-0609^T^	KX710140	KX710145	MF135659	–	Hyde et al. (2016)
*D*. *neorostrata*	MFLUCC 18-0376^T^	MN163017	MN163008	–	–	Luo et al. (2019)
*D*. *nonrostrata*	KUNCC 21-10730^T^	OK341198	OK310699	OP413481	OP413475	Zhang et al. (2022)
*D*. *obclavata*	MFLUCC 18-0329^T^	MN163010	MN163012	–	–	Luo et al. (2019)
*D*. *obpyriformis*	MFLUCC 17-01694^T^	MG979764	–	MG988422	MG988415	Luo et al. (2018)
*D*. *obpyriformis*	DLUCC 0867	MG979765	MG979757	MG988423	MG988416	Luo et al. (2018)
*D*. *pachyconidia*	KUMCC 21-10724^T^	OK341194	OK310696	OP413477	OP413471	Zhang et al. (2022)
***D*. *pachyconidia***	**GZCC 22-0074**	**ON527942**	**ON527934**	**ON533678**	**ON533686**	**This study**
*D*. *palmarum*	MFLUCC 18-1446^T^	MK079663	MK085062	MK087660	MK087670	Hyde et al. (2019)
*D*. *phangngaensis*	MFLUCC 16-0857^T^	MF077556	MF077545	MF135653	–	Yang et al. (2018)
*D*. *rayongensis*	MFLUCC 18-0415^T^	MH457137	MH457172	MH463253	MH463255	Hyde et al. (2020)
*D*. *rayongensis*	MFLUCC 18-0417	MH457138	MH457173	MH463254	MH463256	Hyde et al. (2020)
*D*. *rostrata*	MFLUCC 16-0969^T^	MG979766	MG979758	MG988424	MG988417	Luo et al. (2018)
*D*. *rostrata*	DLUCC 0885	MG979767	MG979759	MG988425	–	Luo et al. (2018)
*D*. *saprophytica*	MFLUCC 18-1238^T^	MW287780	MW286506	MW396651	MW504069	Dong et al. (2021)
***D*. *septata***	**GZCC 22-0078^T^**	**ON527947**	**ON527939**	**ON533683**	**ON533690**	**This study**
*D*. *songkhlaensis*	MFLUCC 18-1234^T^	MW287755	MW286482	MW396642	–	Dong et al. (2021)
*D*. *submersa*	MFLUCC 16-0946^T^	MG979768	MG979760	MG988426	MG988418	Luo et al. (2018)
*D*. *suoluoensis*	MFLUCC 17-0224^T^	MF077557	MF077546	MF135654	–	Yang et al. (2018)
*D*. *suoluoensis*	MFLUCC 17-1305	MF077558	MF077547	–	–	Yang et al. (2018)
*D*. *tectonae*	MFLUCC 12-0291^T^	KX751713	KX751711	KX751710	KX751708	Hyde et al. (2016)
*D*. *tectonae*	MFLU 20-0262	MT232719	MT232714	–	–	Sun et al. (2020)
*D*. *tectonae*	MFLUCC 15-0981	MW287763	MW286489	MW396641	–	Dong et al. (2021)
*D*. *tectonigena*	MFLUCC 12-0292^T^	KX751714	KX751712	–	KX751709	Hyde et al. (2016)
*D*. *thailandica*	MFLUCC 16-0270^T^	MH260292	MH275060	MH412767	–	Tibpromma et al. (2018)
*D*. *thysanolaenae*	KUMCC 18-0182^T^	MK064091	MK045851	MK086031	–	Phukhamsak et al. (2019)
***D*. *tropica***	**GZCC 22-0076^T^**	**ON527943**	**ON527935**	**ON533679**	**ON533687**	**This study**
*D*. *verrucosa*	HKAS 112652^T^	MZ868762	MZ868771	MZ892977	MZ892983	Yang et al. (2021)
***D*. *wuzhishanensis***	**GZCC 22-0077^T^**	**ON527946**	**ON527938**	**ON533682**	**–**	**This study**
*D*. *xishuangbannaensis*	KUMCC 17-0290^T^	MH260293	MH275061	MH412768	MH412754	Tibpromma et al. (2018)
***D*. *xishuangbannaensis***	**GZCC 22-0079**	**ON527944**	**ON527936**	**ON533680**	**ON533688**	**This study**
*D*. *yongxiuensis*	JAUCC 4725^T^	OK562394	OK562388	OK562406	–	Zhai et al. (2022)
*D*. *yunjushanensis*	JAUCC 4723^T^	OK562398	OK562392	OK562410	–	Zhai et al. (2022)
*D*. *yunnanensis*	MFLUCC 20-0153^T^	MW081546	MW081541	MW084995	MW151861	Li et al. (2021)
*Myrmecridium* *aquaticum*	MFLUCC 15-0366^T^	MK849804	–	–	–	Luo et al. (2019)
*M*. *aquaticum*	S 1158	MK849803	MK828656	MN194061	MN124540	Luo et al. (2019)
*M*. *banksiae*	CBS 132536^T^	JX069855	JX069871	–	–	Crous et al. (2012)
*M*. *montsegurinum*	JF 13180^T^	KT991664	KT991674	–	KT991654	Réblová et al. (2016)
*M*. *schulzeri*	CBS 100.54	EU041826	EU041769	–	–	Arzanlou et al. (2007)
*Pseudostanjehughesia* *aquitropica*	MFLUCC 16-0569^T^	MF077559	MF077548	MF135655	–	Yang et al. (2018)
*Ps*. *lignicola*	MFLUCC 15-0352^T^	MK849787	MK828643	MN194047	MN124534	Luo et al. (2019)
*Sporidesmium* *bambusicola*	HKUCC 3578^T^	DQ408562	–	–	–	Shenoy et al. (2006)
*S*. *dulongense*	MFLUCC 17-0116^T^	MH795817	MH795812	MH801191	MH801190	Luo et al. (2019)
*S*. *lageniforme*	DLUCC 0880^T^	MK849782	MK828640	MN194044	MN124533	Luo et al. (2019)
*S*. *pyriformatum*	MFLUCC 15-0620^T^	KX710141	KX710146	MF135662	MF135649	Hyde et al. (2016)
*S*. *thailandense*	MFLUCC 15-0617	MF077561	MF077550	MF135657	–	Yang et al. (2018)
*S*. *thailandense*	MFLUCC 15-0964^T^	MF374370	MF374361	MF370957	MF370955	Zhang et al. (2017)

Note: T denote ex-type strain. Newly generated sequences are indicated in bold. “–”means no data available in GenBank.

## Data Availability

All sequences generated in this study were submitted to GenBank (https://www.ncbi.nlm.nih.gov/genbank/, accessed on 16 May 2022).

## References

[1] Hyde K.D., Bao D.F., Hongsanan S., Chethana K., Yang J., Suwannarach N. (2021). Evolution of freshwater Diaporthomycetidae (Sordariomycetes) provides evidence for five new orders and six new families. Fungal Divers..

[2] Luo Z.L., Hyde K.D., Liu J.K., Maharachchikumbura S.S.N., Jeewon R., Bao D.F., Bhat D.J., Lin C.G., Li W.L., Yang J. (2019). Freshwater Sordariomycetes. Fungal Divers..

[3] Dong W., Hyde K.D., Jeewon R., Doilom M., Yu X.D., Wang G.N., Liu N.G., Hu D.M., Nalumpang S., Zhang H. (2021). Towards a natural classification of annulatascaceae-like taxa II: Introducing five new genera and eighteen new species from freshwater. Mycosphere.

[4] Yang J., Maharachchikumbura S.S.N., Hyde K.D., Bhat D.J., McKenzie E.H.C., Bahkali A.H., Gareth Jones E.B.G., Liu Z.Y. (2015). *Aquapteridospora lignicola* gen. et sp. nov., a new hyphomycetous taxon (Sordariomycetes) from wood submerged in a freshwater stream. Cryptogam. Mycol..

[5] Wijayawardene N.N., Hyde K.D., Al-Ani L.K.T., Tedersoo L., Haelewaters D., Rajeshkumar K.C., Zhao R.L., Aptroot A., Leontyev D.V., Saxena R.K. (2020). Outline of Fungi and fungus-like taxa. Mycosphere.

[6] Dai D.Q., Phookamsak R., Wijayawardene N.N., Li W.J., Bhat D.J., Xu J.C., Taylor J.E., Hyde K.D., Chukeatirote E. (2016). Bambusicolous fungi. Fungal Divers..

[7] Yang J., Liu L.L., Jones E.B.G., Li W.L., Hyde K.D., Liu Z.Y. (2021). Morphological variety in *Distoseptispora* and introduction of six novel species. J. Fungi..

[8] Bao D.F., Hyde K.D., McKenzie E.H.C., Jeewon R., Su H.Y., Nalumpang S., Luo Z.L. (2021). Biodiversity of lignicolous freshwater hyphomycetes from China and Thailand and description of sixteen species. J. Fungi..

[9] Su H.Y., Hyde K.D., Maharachchikumbura S., Ariyawansa H.A., Luo Z.L., Promputtha I., Tian Q., Lin C.G., Shang Q.J., Zhao Y.C. (2016). The families Distoseptisporaceae fam. nov., Kirschsteiniotheliaceae, Sporormiaceae and Torulaceae, with new species from freshwater in Yunnan Province, China. Fungal Divers..

[10] Yang J., Maharachchikumbura S., Liu J.K., Hyde K.D., Jones E.B.G., Al-Sadi A.M., Liu Z.Y. (2018). *Pseudostanjehughesia aquitropica* gen. et sp. nov. and *Sporidesmium sensu lato* species from freshwater habitats. Mycol. Prog..

[11] Monkai J., Boonmee S., Ren G., Wei D., Phookamsak R., Mortimer P.E. (2020). *Distoseptispora hydei* sp. nov. (Distoseptisporaceae), a novel lignicolous fungus on decaying bamboo in Thailand. Phytotaxa.

[12] Shen H.W., Bao D.F., Hyde K.D., Su H.Y., Bhat D.J., Luo Z.L. (2021). Two novel species and two new records of *Distoseptispora* from freshwater habitats in China and Thailand. MycoKeys.

[13] Xia J.W., Ma Y.R., Li Z., Zhang X.G. (2017). *Acrodictys*-like wood decay fungi from southern China, with two new families Acrodictyaceae and Junewangiaceae. Sci. Rep..

[14] Hyde K.D., Hongsanan S., Jeewon R., Bhat D.J., McKenzie E.H.C., Jones E.B.G., Phookamsak R., Ariyawansa H., Boonmee S., Zhao Q. (2016). Fungal diversity notes 367–490: Taxonomic and phylogenetic contributions to fungal taxa. Fungal Divers..

[15] Hyde K.D., Tennakoon D.S., Jeewon R., Bhat D.J., Maharachchikumbura S., Rossi W., Leonardi M., Lee H.B., Mun H.Y., Houbraken J. (2019). Fungal diversity notes 1036–1150: Taxonomic and phylogenetic contributions on genera and species of fungal taxa. Fungal Divers..

[16] Zhang H., Zhu R., Qing Y., Yang H., Li C.X., Wang G.N., Zhang D., Ning P. (2022). Polyphasic identification of *Distoseptispora* with six new species from freshwater. J. Fungi.

[17] Maharachchikumbura S.S.N., Hyde K.D., Jones E.B.G., McKenzie E.H.C., Bhat D.J., Dayarathne M.C., Huang S.K., Norphanphoun C., Senanayake I.C., Perera R.H. (2016). Families of Sordariomycetes. Mycosphere.

[18] Luo Z.L., Hyde K.D., Liu J.K., Bhat D.J., Su H., Bao D.F., Li W.L. (2018). Lignicolous freshwater fungi from China II: Novel *Distoseptispora* (Distoseptisporaceae) species from northwestern Yunnan Province and a suggested unified method for studying lignicolous freshwater fungi. Mycosphere.

[19] Phookamsak R., Hyde K.D., Jeewon R., Bhat D.J., Jones E.B.G., Maharachchikumbura S., Raspé O., Karunarathna S.C., Wanasinghe D., Hongsanan S. (2019). Fungal diversity notes 929–1035: Taxonomic and phylogenetic contributions on genera and species of fungi. Fungal Divers..

[20] Tibpromma S., Hyde K.D., Mckenzie E., Bhat D.J., Phillips A.J.L., Wanasinghe D.N., Samarakoon M.C., Jayawardena R.S. (2018). Fungal diversity notes 840–928: Micro-fungi associated with Pandanaceae. Fungal Divers..

[21] Li W.L., Liu Z.P., Zhang T., Dissanayake A.J., Luo Z.L., Su H.Y., Liu J.K. (2021). Additions to *Distoseptispora* (Distoseptisporaceae) associated with submerged decaying wood in China. Phytotaxa.

[22] Shoemaker R.A., White G.P. (1985). *Lasiosphaeria caesariata* with *Sporidesmium hormiscioides* and *L. triseptata* with *S. adscendens*. Sydowia.

[23] McKenzie E.H.C. (1995). Dematiaceous hyphomycetes on Pandanaceae. 5. Sporidesmium sensu lato. Mycotaxon.

[24] Hyde K.D. (2021). Mycosphere notes 325-344–Novel species and records of fungal taxa from around the world. Mycosphere.

[25] Sun Y., Goonasekara I.D., Thambugala K.M., Jayawardena R.S., Wang Y., Hyde K.D. (2020). *Distoseptispora bambusae* sp. nov. (Distoseptisporaceae) on bamboo from China and Thailand. Biodivers. Data J..

[26] Phukhamsakda C., McKenzie E.H.C., Phillips A., Jones E.B.G., Bhat D.J., Stadler M., Bhunjun C.S., Wanasinghe D.N., Thongbai B., Camporesi E. (2020). Microfungi associated with *Clematis* (Ranunculaceae) with an integrated approach to delimiting species boundaries. Fungal Divers..

[27] Phukhamsakda C., Nilsson R.H., Bhunjun C.S., Farias A.R.G.D., Sun Y., Wijesinghe S.N., Raza M., Bao D.F., Lu L., Tibpromma S. (2022). The numbers of fungi: Contributions from traditional taxonomic studies and challenges of metabarcoding. Fungal Divers..

[28] Zhai Z.J., Yan J.Q., Li W.W., Gao Y., Hu H.J., Zhou J.P., Song H.Y., Hu D.M. (2022). Three novel species of *Distoseptispora* (Distoseptisporaceae) isolated from bamboo in Jiangxi Province, China. MycoKeys.

[29] Crous P., Wingfield M., Lombard L., Roets F., Swart W., Alvarado P., Carnegie A., Moreno G., Luangsa-Ard J., Thangavel R. (2019). Fungal Planet description sheets: 951–1041. Pers. Mol. Phylogeny Evol. Fungi..

[30] Senanayake I.C., Rathnayaka A.R., Marasinghe D.S., Calabon M.S., Gentekaki E., Lee H.B., Hurdeal V.G., Pem D., Dis-sanayake L.S., Wijesinghe S.N. (2020). Morphological approaches in studying fungi: Collection, examination, isolation, sporulation and preservation. Mycosphere.

[31] Jayasiri S.C., Hyde K.D., Ariyawansa H.A., Bhat J., Buyck B., Cai L., Dai Y.C., Abd-Elsalam K.A., Ertz D., Hidayat I. (2015). The Faces of Fungi database: Fungal names linked with morphology, phylogeny and human impacts. Fungal Divers..

[32] Index Fungorum. http://www.indexfungorum.org/names/names.asp..

[33] Vilgalys R., Hester M. (1990). Rapid genetic identification and mapping of enzymatically amplified ribosomal DNA from several *Cryptococcus* species. J. Bacteriol..

[34] White T., Bruns T., Lee S., Taylor J., Innis M.A., Gelfand D.H., Sninsky J.J., White T.J. (1990). Amplification and direct sequencing of fungal ribosomal RNA genes for phylogenetics. PCR Protocols—A Guide to Methods and Applications.

[35] Rehner S.A., Samuels G.J. (1994). Taxonomy and phylogeny of *Gliocladium* analysed from nuclear large subunit ribosomal DNA sequences. Mycol. Res..

[36] Liu Y.J., Whelen S., Hall B.D. (1999). Phylogenetic relationships among ascomycetes: Evidence from an RNA polymerse II subunit. Biol. Evol..

[37] Hall T.A. (1999). BioEdit: A user-friendly biological sequence alignment editor and analysis program for windows 95/98/NT. Nucleic Acids Symp. Ser..

[38] Katoh K., Standley D.M. (2013). Evolution. MAFFT multiple sequence alignment software version 7: Improvements in performance and usability. Biol. Evol..

[39] Capella-Gutiérrez S., Silla-Martínez J.M., Gabaldón T. (2009). trimAl: A tool for automated alignment trimming in large-scale phylogenetic analyses. Bioinformatics.

[40] Daniel G.P., Daniel G.B., Miguel R.J., Florentino F.R., David P. (2010). ALTER: Program-oriented conversion of DNA and protein alignments. Nucleic Acids Res..

[41] Miller M.A., Pfeiffer W., Schwartz T. Creating the CIPRES Science Gateway for inference of large phylogenetic trees. Proceedings of the 2010 Gateway Computing Environments Workshop (GCE).

[42] Stamatakis A. (2014). RAxML version 8: A tool for phylogenetic analysis and post-analysis of large phylogenies. Bioinformatics.

[43] Swofford D.L. (2003). PAUP*. Phylogenetic Analysis Using Parsimony (*and Other Methods).

[44] Hillis D.M., Bull J.J. (1993). An Empirical Test of Bootstrapping as a Method for Assessing Confidence in Phylogenetic Analysis. Syst. Biol..

[45] Nylander J.A.A., Zoology S., Posada D., Mrmodeltest R., Os F. (2008). MrModeltest2 v. 2.3 (Program for Selecting DNA Substitution Models Using PAUP*).

[46] Huelsenbeck J.P., Ronquist F.J.B. (2001). MRBAYES: Bayesian inference of phylogenetic trees. Bioinformatics.

[47] Zhang H., Dong W., Hyde K.D., Maharachchikumbura S.S., Hongsanan S., Bhat D.J., Al-Sadi A.M., Zhang D. (2017). Towards a natural classification of *Annulatascaceae*-like taxa: Introducing Atractosporales ord. nov. and six new families. Fungal Divers..

[48] Crous P.W., Summerell B.A., Shivas R.G., Burgess T.I., Decock C.A., Dreyer L.L., Granke L.L., Guest D.I., Hardy G., Hausbeck M. (2012). Fungal Planet description sheets: 107–127. Persoonia.

[49] Arzanlou M., Groenewald J.Z., Gams W., Braun U., Shin H.D., Crous P.W. (2007). Phylogenetic and morphotaxonomic revision of *Ramichloridium* and *allied* genera. Stud. Mycol..

[50] Réblová M., Fournier J., Štěpánek V. (2016). Two new lineages of aquatic ascomycetes: *Atractospora* gen. nov. and *Rubellisphaeria* gen. et sp. nov., and a sexual morph of *Myrmecridium montsegurinum* sp. nov. Mycol. Prog..

[51] Chethana K.W.T., Manawasinghe I.S., Hurdeal V.G., Bhunjun C.S., Appadoo M.A., Gentekaki E., Raspé O., Promputtha I., Hyde K.D. (2021). What are fungal species and how to delineate them?. Fungal Divers..

[52] Maharachchikumbura S.S.N., Chen Y., Ariyawansa H.A., Hyde K.D., Haelewaters D., Perera R.H., Samara-koon M.C., Wanasinghe D.N., Bustamante D.E., Liu J.K. (2021). Integrative approaches for species delimitation in Ascomycota. Fungal Divers..

[53] Pem D., Jeewon R., Chethana K.W.T., Hongsanan S., Doilom M., Suwannarach N., Hyde K.D. (2021). Species concepts of Dothideomycetes: Classification, phylogenetic inconsistencies and taxonomic standardization. Fungal Divers..

[54] Shenoy B.D., Jeewon R., Wu W.P., Bhat D.J., Hyde K.D. (2006). Ribosomal and RPB2 DNA sequence analyses suggest that *Sporidesmium* and morphologically similar genera are polyphyletic. Mycol. Res..

[55] Bhunjun C.S., Niskanen T., Suwannarach N., Wannathes N., Chen Y.-J., McKenzie E.H.C., Maharachchikumbura S.S.N., Buyck B., Zhao C.-L., Fan Y.-G. (2022). The numbers of fungi: Are the most speciose genera truly diverse?. Fungal Divers..

[56] Hyde K.D., Jeewon R., Chen Y.J., Bhunjun C.S., Calabon M.S., Jiang H.B., Lin C.G., Norphanphoun C., Sysouphanthong P., Pem D. (2020). The numbers of fungi: Is the descriptive curve flattening?. Fungal Divers..

[57] Dong W., Wang B., Hyde K.D., McKenzie E.H.C., Raja H.A., Tanaka K., Abdel-Wahab M.A., Abdel-Aziz F.A., Doilom M., Phookamsak R. (2020). Freshwater Dothideomycetes. Fungal Divers..

[58] Peng S.Q., Liu Y.L., Huang J.E., Li X.H., Yan X.Y., Song H.Y., Gao Y., Zhai Z.J., Liu Y.Q., Hu D.M. (2022). *Aquapteridospora jiangxiensis*, a new aquatic hyphomycetous fungus from a freshwater habitat in China. Arch. Microbiol..

